# Anti-COX-2 autoantibody is a novel biomarker of immune aplastic anemia

**DOI:** 10.1038/s41375-022-01654-6

**Published:** 2022-08-05

**Authors:** Tiina Kelkka, Mikko Tyster, Sofie Lundgren, Xingmin Feng, Cassandra Kerr, Kohei Hosokawa, Jani Huuhtanen, Mikko Keränen, Bhavisha Patel, Toru Kawakami, Yuka Maeda, Otso Nieminen, Tiina Kasanen, Pasi Aronen, Bhagwan Yadav, Hanna Rajala, Hideyuki Nakazawa, Taina Jaatinen, Eva Hellström-Lindberg, Seishi Ogawa, Fumihiro Ishida, Hiroyoshi Nishikawa, Shinji Nakao, Jaroslaw Maciejewski, Neal S. Young, Satu Mustjoki

**Affiliations:** 1 Hematology Research Unit Helsinki, University of Helsinki and Department of Hematology, Helsinki University Hospital Comprehensive Cancer Center, Helsinki, Finland; 2 Translational Immunology Research Program and Department of Clinical Chemistry and Hematology, University of Helsinki, Helsinki, Finland; 3 Hematology Branch, National Heart, Lung, and Blood Institute, NIH, Bethesda, MD USA; 4 Department of Translational Hematology and Oncology Research and Leukemia Program, Department of Hematology and Medical Oncology, Taussig Cancer Institute, Cleveland Clinic, Cleveland, OH USA; 5 Department of Hematology, Faculty of Medicine, Institute of Medical Pharmaceutical and Health Sciences, Kanazawa University, Kanazawa, Japan; 6 Division of Hematology, Department of Internal Medicine, Shinshu University School of Medicine, Matsumoto, Japan; 7 Division of Cancer Immunology, Research Institute/Exploratory Oncology Research and Clinical Trial Center, National Cancer Center Japan, Tokyo, Japan; 8 Biostatistics Unit, Faculty of Medicine, University of Helsinki and Helsinki-Uusimaa Hospital District, Helsinki, Finland; 9 Department of Hematology, Shinshu University School of Medicine, Matsumoto, Japan; 10 Histocompatibility Testing Laboratory, Finnish Red Cross Blood Service, Helsinki, Finland; 11 Division of Hematology, Department of Medicine, Karolinska University Hospital, Stockholm, Sweden; 12 Department of Pathology and Tumor Biology, Graduate School of Medicine, Kyoto University, Kyoto, Japan; 13 Department of Biomedical Laboratory Sciences, Shinshu University School of Medicine, Matsumoto, Japan; 14 iCAN Digital Precision Cancer Medicine Flagship, Helsinki, Finland

**Keywords:** Autoimmune diseases, Anaemia, Antibodies

## Abstract

In immune aplastic anemia (IAA), severe pancytopenia results from the immune-mediated destruction of hematopoietic stem cells. Several autoantibodies have been reported, but no clinically applicable autoantibody tests are available for IAA. We screened autoantibodies using a microarray containing >9000 proteins and validated the findings in a large international cohort of IAA patients (*n* = 405) and controls (*n* = 815). We identified a novel autoantibody that binds to the C-terminal end of cyclooxygenase 2 (COX-2, aCOX-2 Ab). In total, 37% of all adult IAA patients tested positive for aCOX-2 Ab, while only 1.7% of the controls were aCOX-2 Ab positive. Sporadic non-IAA aCOX-2 Ab positive cases were observed among patients with related bone marrow failure diseases, multiple sclerosis, and type I diabetes, whereas no aCOX-2 Ab seropositivity was detected in the healthy controls, in patients with non-autoinflammatory diseases or rheumatoid arthritis. In IAA, anti-COX-2 Ab positivity correlated with age and the HLA-DRB1*15:01 genotype. 83% of the >40 years old IAA patients with HLA-DRB1*15:01 were anti-COX-2 Ab positive, indicating an excellent sensitivity in this group. aCOX-2 Ab positive IAA patients also presented lower platelet counts. Our results suggest that aCOX-2 Ab defines a distinct subgroup of IAA and may serve as a valuable disease biomarker.

## Introduction

Aplastic anemia (AA) is a rare bone marrow failure (BMF) disease characterized by the loss of all hematopoietic cell lineages (pancytopenia) [1]. The disease develops via three alternate routes: chemical/physical insults (including radiation and toxic agents), hereditary genetic defects or via immune-mediated mechanisms. Sporadic AA cases without a family history or documented chemical exposure are considered as immune-mediated (IAA), and they are the largest patient group. AA treatments include allogeneic bone marrow transplantation, the use of immunosuppressive therapy (IST) (anti-thymocyte globulins, cyclosporin A) and growth factor receptor agonists (eltrombopag, granulocyte-colony stimulating factor). AA patients encounter life-threatening cytopenias, and up to 15% [2–4] of patients develop myelodysplastic syndrome (MDS) or ultimately acute myeloid leukemia.

Adult IAA is associated with the HLA class II DRB1 antigen 15 (alleles *15:01, *15:02) [5]. In pediatric IAA, the associated HLA alleles differ [6] suggesting different immune pathomechanisms in different age groups. HLA-DRB1*15:01 is also linked to better treatment responses to cyclosporin A [5] and to the presence of the PNH clone (cells deficient of glycophosphoinositol-anchored proteins) [7]. IAA patients with a PNH clone respond better to IST [8]. In contrast, increasing age is associated with worse prognosis; less than 40% of >60 years old IAA patients are alive 5 years after the diagnosis [9].

The pathogenesis of IAA is not understood in detail. Roughly two thirds of the patients respond to IST [1], which emphasizes the immune-mediated mechanisms in IAA. Cytotoxic T cells display an activated phenotype [10, 11], and CD4+ regulatory T cells (Tregs) are decreased, thus assigning T cells as important regulators of IAA [12]. The importance of non-hematopoietic cells is highlighted by the recent finding showing that bone marrow mesenchymal stem cells can regulate Treg/Th17 balance in IAA [13]. Somatic gene and chromosome aberrations leading to the loss of one HLA I haplotype [14–19] are additional factors modulating the HSCs susceptibility to immune attack, likely by reducing recognition and attack by autoreactive T cells [16, 20].

Repeated autoantibody findings point to the involvement of the humoral immunity in the induction and/or maintenance of autoimmunity in IAA. Previously, IAA-associated autoantibodies have been detected against kinectin [21, 22], diazepam-binding inhibitor-related protein 1 (DRS-1) [23], carbonic anhydrase 1 (CA-1) [24, 25], heterogeneous nuclear ribonucleoprotein (hnRNP) K [26], chloride intracellular channel 1 (CLIC1), heat shock binding protein (HSBP11), ribosomal protein S27 (RPS27) [27], moesin [28] and postmeiotic segregation increased 1 (PMS-1) [22]. Most of these antibodies are not specific for IAA, but are also detected in other autoimmune, BMF or inflammatory diseases.

BMF diseases are challenging to diagnose due to overlapping phenotypes and gradual transitions from one condition to another. AA diagnosis is a rule-out diagnosis, based on bone marrow morphology, peripheral blood counts, cytogenetics and genetic deep sequencing analyses including both germline and somatic variant analyses [29]. Here, we identified a novel anti-prostaglandin G/H synthase 2 or cyclooxygenase-2 (COX-2) autoantibody (aCOX-2 Ab) that is associated with IAA. In a large international cohort of IAA patients (*n* = 405) we confirmed that the autoantibody not only associates with the HLA-DRB1*15:01 genotype but also with older age, and lower platelet counts at diagnosis.

## Methods

### Patient samples

All analysis were performed using plasma (EDTA or heparin anticoagulated) or serum samples from patients and controls depending on availability. The DELFIA assay has been confirmed to perform well with all these sample formats. Out of the total of 405 IAA patients, 334 were adults (>18 years old) and 276 had sufficient clinical data coverage to be included in the regression analysis. All IAA patients were diagnosed according to routine clinical procedures by experienced clinicians. In addition, we analyzed plasma samples from 815 controls (Supplementary Table 1). In total, 409 of the control samples was obtained from Helsinki Biobank (Supplementary Table 2).

### Autoantibody screen

Autoantibodies were screened from peripheral plasma samples using the Invitrogen (Carlsbad, CA) ProtoArray protein microarray v.5.1 (https://www.thermofisher.com/jp/en/home/life-science/protein-biology/protein-assays-analysis/protein-microarrays.html) as previously described [30]. The cutoff for positivity was set to Fold Change 10 compared to the average of healthy controls.

### DELFIA assays

A recombinant cyclooxygenase-2 protein encoded by the *PTGS2* gene (Sino Biological Inc, Beijing, China, Cat. No. 12036-H08B) was used in the binding studies. The protein consisted of amino acids 1-604 of the human COX-2, was C-terminally His-tagged and supplied in frozen solution form.

Antibody binding studies were carried out with sandwiched Dissociation Enhanced Lanthanide Fluorescence Immunoassays (DELFIA). Each well of a 96-well Nunc-Immuno Maxisorp plate (Sigma Aldrich, Saint Louis, MO, Cat. No. M5785-1CS) was coated with 250 ng of mouse anti-His-tag antibody (Thermo Fisher Scientific, Rockford, IL, Cat. No. MA1-135) in phosphate-buffered saline (PBS; Corning Life Sciences, Oneonta, NY, Cat. No. 15313581) overnight in room temperature (RT). The plates were washed in four cycles with PBS + 0.05% Tween 20 (PBS-T) using DELFIA Platewash (PerkinElmer, Shelton, CT). Wells were blocked against non-specific protein binding with 1% DTPA-purified bovine serum albumin (BSA; PerkinElmer) for 1 h in RT. After washing, wells were incubated with 100 ng/well of recombinant COX-2 protein in a diluting buffer (PBS-T with 0.2% of DTPA-purified BSA; PBS-T + BSA) for 1 h in RT. After wash cycles, plasma/serum samples were added diluted 1:100 in the diluting buffer (PBS-T + BSA) in duplicates. Blank controls were included in duplicate on each plate. Each set of plates included a series of 6 standards prepared from a cross-reacting rabbit anti-human-COX-2 antibody (SDIX LLC, Newark, DE, Cat. No. 23240002). After incubating for 1 h in RT and washing for four cycles 100 µl/well of Eu-labeled mouse anti-human-IgG antibody (PerkinElmer, Cat. No. 1244-330) diluted 1:1000 in DELFIA Assay buffer (PerkinElmer, Cat. No. 1244-111) was added and incubated for 1 h in RT. The Eu-labeled detection antibody was washed off for six cycles, DELFIA Enhancement Solution (PerkinElmer, Cat. No. 1244-105) was added, and plates incubated for 5 min before fluorescence data acquisition with Victor X4 plate reader (PerkinElmer) with Time-resolved Fluorometry Europium protocol (excitation at 340 nm).

IgG subclass isotypes IgG_1_–IgG_4_ were determined with the same DELFIA method as total IgG aCOX-2 Ab. Instead of the Eu-labeled anti-human-IgG antibody, subclass-specific biotinylated mouse anti-human-IgG_1_–IgG_4_ (Sigma Aldrich, Saint Louis, MO, Cat. No. B6775, B3398, B3523, B3648) were used. Anti-IgG_1_ was diluted 1:1000, anti-IgG_2_ and anti-IgG_3_ 1:5000 and anti-IgG_4_ 1:10,000 in DELFIA Assay buffer.

For the detection of anti-COX-2 IgA and IgM isotypes the Eu-labeled anti-human-IgG antibody, was replaced with a biotinylated goat anti-human-IgA (α chain) or -IgM (µ chain) antibody (Thermo Fisher Scientific, Rockford, IL, Cat. No. A18791 and A18845). Both antibodies were diluted 1:7500 in DELFIA Assay buffer and used 100 µl/well. These were followed by Eu-labeled streptavidin 1:1000 in DELFIA Assay buffer as described above for IgG subclass isotypes.

### Determination of test positivity threshold

To separate positive cases from negative two *R* packages Findcutoffs [31] and OptimalCutPoint [32] were used. The combined measurements of 681 patient samples (all cohorts excluding the controls from Helsinki Biobank) were used as training data, and the 300 Helsinki Biobank samples were used as the validation group. Likelihood ratio test for statistical significance and AUC were used for the former package and Youden’s index and AUC for the latter as optimization methods. As the resulting cutoff levels were very close to each other (Supplementary Fig. 1), their mean value was chosen as the cutoff for positivity.

### Epitope mapping

Both linear and conformational anti-COX-2 Ab binding COX-2 (UniProt ID P35354) epitopes were mapped with the commercially available PEPperPRINT® technology (technical details in Supplementary Methods).

### Western blot

Recombinant COX-2 (Sino Biological Inc, Beijing, China, Cat. No. 12036-H08B) was mixed with Laemmli buffer (Bio-Rad Laboratories, Hercules, CA), PBS and DTT (Dithiothreitol, Merck, Cat. No. 646563) to a final concentration of 2.5 μg/ml. In total, 20 μl of this solution was applied on a 7.5% SDS-PAGE precast gel (Bio-Rad Laboratories, Cat. No. 4561025) together with WesternSure Pre-stained Chemiluminescent Protein Ladder (LI-COR Biosciences, Lincoln, NE, Cat. No. 926-98000) and the proteins were transferred to a nitrocellulose membrane (Bio-Rad Laboratories, Hercules, CA, Cat. No. 1704270). Odyssey blocking buffer (OBB, LI-COR Biosciences, Cat. No. 927-40000) mixed 1:1 with PBS was used as blocking solution before incubation with patient plasma diluted (1:2000) in 60% PBS, 40% OBB and 0.2% Tween 20. Mouse anti-human IgG, Fc Fragment Specific (HP6043) Peroxidase Conjugate (1:1000) (Merck Millipore, Burlington, MA, Cat. No. 411550), diluted in 60% PBS, 40% OBB and 0.2% Tween 20 was used to detect the autoantibodies using Clarity Western ECL Blotting Substrates (Bio-Rad Laboratories) and ChemiDoc MP Imaging System (Bio-Rad Laboratories).

### Single-cell RNA-sequencing data analysis

Single-cell RNA-sequencing data from sorted CD34+ cells from bone marrow samples from AA patients (*n* = 15) and healthy donors (*n* = 2) were gathered from Zhu et al. [33]. After quality control (mitochondrial transcripts <10%, ribosomal transcripts <50%, number of genes between 500 and 3000 per cell, number of UMI reads between 1000 and 30,000 counts), the data were log-normalized (scaling factor of 10,000) and scaled using the genes with the highest variance (top 2000) with Seurat [34] (3.0.0). To overcome batch effect, scVI [35] (0.5.0) with default parameters was used to calculate latent embeddings, which were then used for subsequent graph-based clustering and UMAP dimensionality reduction with Seurat with default parameters. Cell clusters were annotated with an ensemble method including analysis of canonical marker genes, calculating the most differentially expressed genes, and analysis with reference-based method SingleR [36] (1.2.4) performed with default parameters.

### Statistical methods

Logistic regression analysis was performed with HLA-DRB*15:01, gender, age, PNH clone, IAA severity, diagnostic phase hemoglobin, white blood cell, platelet, absolute neutrophil count and absolute lymphocyte count values as independent variables explaining the presence of aCOX-2 Ab. In addition, we squared the “age at diagnosis” to check for linearity. Data from all >18 years old IAA patients, with <30% missing values (patterns of missing values are presented in Supplementary Fig. 2) and with HLA-DRB1*15 and treatment variables available were included in the logistic regression analysis. MICE package [37] using the predictive mean matching was used to impute the ten included datasets. Regression analyses and model validation (Dharma-package) were performed using the R Software.

GraphPad Prism 9 software (GraphPad Software, La Jolla, CA, USA) was used to produce other statistical analyses and illustrations.

## Results

### Identification of anti-COX2 antibody in IAA patients

**Fig. 1:**
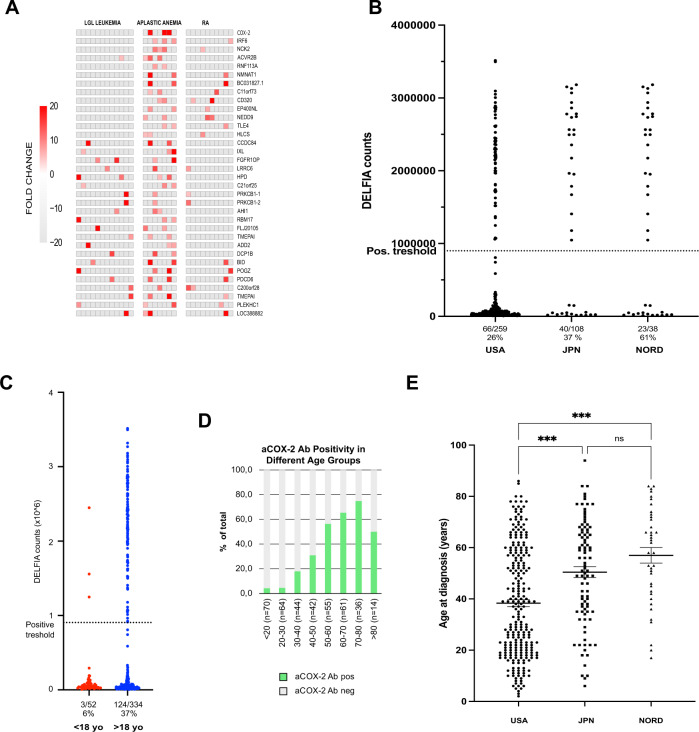
Identification of autoantibodies using a protein microarray platform and aCOX-2 antibodies in different IAA cohorts. **A** Autoantibody screening results from patients with large granular lymphocyte (LGL) leukemia (*n* = 12), immune aplastic anemia (*n* = 7) and rheumatoid arthritis (*n* = 10) were compared to the mean values from healthy controls (HC, *n* = 5). All values that were more than ten times higher than the mean of fold change of HC were considered as positive. Heat map presents data from all individual antibodies that were positive in at least two individual AA patients. RA Rheumatoid Arthritis. **B** Dashed line denotes the statistical cutoff value determined from combined cohort data. aCOX-2 Ab results expressed as DELFIA counts from the United States (USA), Japan (JPN) and Nordic (NORD) IAA patient cohorts. **C** Percentages of aCOX-2 positive in pediatric (<18 years old) and adult (>18 years old) IAA patients. **D** Percentages of aCOX-2 Ab positive IAA patients in age groups divided into 10-year intervals. **E** Median ages at diagnosis and interquartile ranges are presented for all IAA patients included in the USA, Japanese (JPN) and Nordic (NORD) patient cohorts. ANOVA was used for comparisons between the groups.

Protoarray protein microarray analysis was performed to identify potential autoantibodies in patients with IAA (*n* = 7), large granular lymphocyte (LGL) leukemia (*n* = 12), rheumatoid arthritis (RA) (*n* = 10) and healthy controls (*n* = 5). Figure 1A presents all protein targets of autoantibodies that were present in at least two of the tested IAA patients. COX-2 was the only protein with IAA restricted autoantibody levels with >20-fold difference to healthy controls in all positive cases.

Next, we developed an aCOX-2 IgG DELFIA immunoassay to confirm the microarray results. The DELFIA immunoassay results were in accordance with the protein microarray data, and aCOX-2 antibodies were confirmed in all three index cases but in none of the negative control patients (Supplementary Fig. 3).

To validate the findings in a larger patient cohort, we collected an international IAA cohort including a total of 405 patients (US *n* = 259, Japan *n* = 108, and Nordic countries *n* = 38). Sample positivity threshold for aCOX-2 Ab was set to correspond the turning point in ROC curve maximizing test sensitivity (0.36) and specificity (Supplementary Fig. 1). The highest aCOX-2 Ab positivity (61%) was found in the Nordic cohort, while in the US and Japanese IAA cohorts there were 26% and 37% aCOX-2 Ab positive patients, respectively (Fig. 1B). aCOX-2 Ab positive patients were mostly adults (Fig. 1C) and when the IAA patient cohort was split in 10-year age intervals, a clear age dependent increase in aCOX-2 Ab positivity was observed especially in patients over 40 years of age (Fig. 1D). Interestingly, the age distribution in different international cohorts followed the aCOX-2 Ab positivity percentages as patients in the Nordic cohort were the oldest, and patients in the USA cohort were the youngest (Fig. 1E). As only sporadic aCOX-2 Ab positive cases were observed in the pediatric patients, only adult (>18 years old) patients (*n* = 334) were included in further analyses. The overall aCOX-2 Ab positivity in adult IAA patients was 37%. Descriptive statistics for the clinical parameters for adult IAA patients (*n* = 334) are presented in Table 1, while the entire cohort (*n* = 405) including the pediatric patients is presented in Supplementary Table 3.

**Table 1:** Study cohort demographics (adult IAA patients *n* = 334).

	aCOX-2 Ab negative	aCOX-2 Ab positive	Total	*p* value
	(*n* = 209)	(*n* = 125)	(*n* = 334)	
Age at diagnosis (years)				
Mean (SD)	39.8 (17.7)	61.8 (13.2)	48.1 (19.4)	**<0.001**
Range	18.0–86.0	20.0–94.0	18.0–94.0	
Gender				
Male	49% (*n* = 102)	42% (*n* = 52)	46% (*n* = 154)	0.2137 (f)
Female	51% (*n* = 107)	58% (*n* = 73)	54% (*n* = 180)	
PNH clone				
Present	49% (*n* = 85)	63% (*n* = 69)	54% (*n* = 154)	**0.0279 (f)**
Absent	51% (*n* = 88)	37% (*n* = 41)	46% (*n* = 129)	
Severity				
Moderate	32% (*n* = 56)	29% (*n* = 30)	31% (*n* = 86)	0.6181 (c)
Severe	61% (*n* = 106)	61% (*n* = 63)	61% (*n* = 169	
Very severe	7% (*n* = 13)	11% (*n* = 11)	9% (*n* = 24)	
Hb at diagnosis (g/dl)				
Mean (SD)	9.03 (2.17)	9.51 (9.68)	9.21 (6.22)	0.514
Range	2.60–16.30	2.80–111.00	2.60–111.00	
WBC at diagnosis (×10^9^/l)				
Mean (SD)	2.49 (1.55)	2.51 (1.23)	2.50 (1.44)	0.940
Range	0.04–9.18	0.08–8.33	0.04–9.18	
Plt at diagnosis (×10^9^/l)				
Mean (SD)	43.38 (48.58)	20.97 (16.54)	34.82 (40.97)	**<0.001**
Range	2–305	1–67	1–305	
ANC at diagnosis (×10^9^/l)				
Mean (SD)	1.00 (0.97)	0.84 (0.72)	0.94 (0.88)	0.137
Range	0.00–5.39	0.01–3.90	0.00–5.39	
ALC at diagnosis (×10^9^/l)				
Mean (SD)	1.25 (0.79)	1.41 (0.71)	1.31 (0.76)	0.123
Range	0.03–4.51	0.28–3.64	0.03–4.51	
ARC at diagnosis (×10^9^/l)				
Mean (SD)	36.44 (38.91)	31.55 (21.88)	34.45 (33.05)	0.316
Range	0.003–374.00	2.00–116.00	0.003–374.00	
Hb at sampling (g/dl)				
Mean (SD)	9.57 (2.07)	9.69 (1.83)	9.61 (1.99)	0.643
Range	3.40–15.10	3.50–15.50	3.40–15.50	
WBC at sampling (×10^9^/l)				
Mean (SD)	2.46 (1.61)	2.91 (3.61)	2.62 (2.50)	0.161
Range	0.03–10.35	0.060–30.30	0.03–30.30	
Plt at sampling (×10^9^/l)				
Mean (SD)	51.41 (62.07)	32.28 (41.54)	44.69 (56.39)	**0.009**
Range	2–511	1–277	1–511	
ANC at sampling (×10^9^/l)				
Mean (SD)	1.15 (1.18)	1.24 (1.80)	1.18 (1.42)	0.608
Range	0.00–8.84	0.00–10.76	0.00–10.76	
ALC at sampling (×10^9^/l)				
Mean (SD)	1.17 (0.75)	1.31 (0.85)	1.22 (0.79)	0.162
Range	0.01–4.30	0.01–5.15	0.01–5.15	
ARC at sampling (×10^9^/l)				
Mean (SD)	33.63 (32.46)	29.03 (21.61)	32.05 (29.17)	0.411
Range	0.003–139.00	0.004–80.16	0.003–139.00	

*p* values are calculated using the linear ANOVA model except for gender, PNH clone and severity which have been calculated using either Fisher’s exact (f) or Chi-square test (c).

*SD* standard deviation, *PNH* paroxysmal nocturnal hemoglobinuria, *Hb* hemoglobin, *WBC* white blood cells, *Plt* platelets, *ANC* absolute neutrophil count, *ALC* absolute lymphocyte count, *ARC* absolute reticulocyte count.

Statistically significant *p*-values are in bold.

### Anti-COX-2 autoantibodies are rarely observed in other patient cohorts

**Fig. 2:**
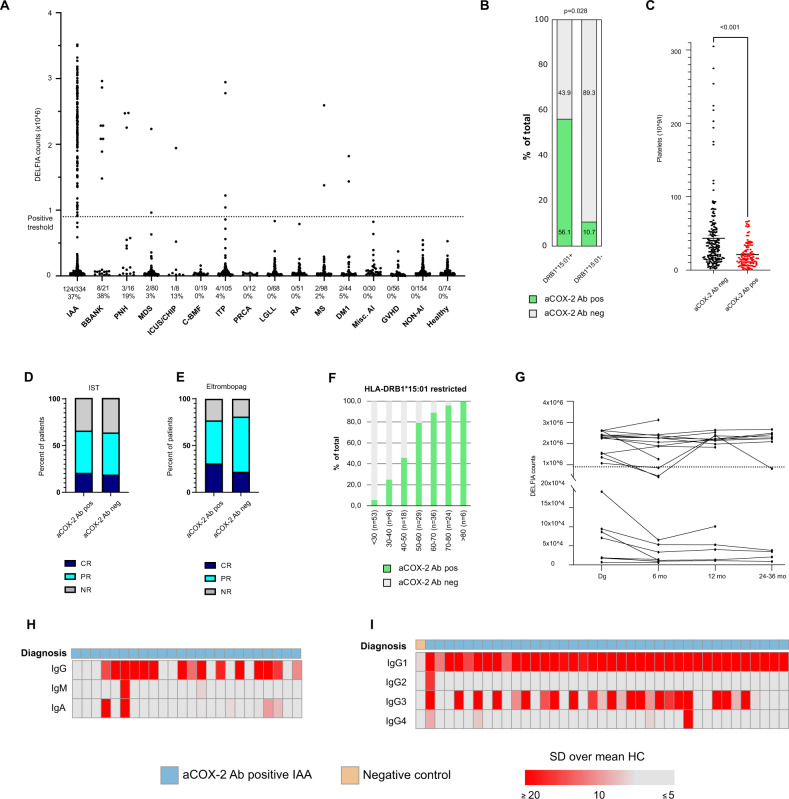
Measurements of aCOX-2 Ab in patient plasma by DELFIA. **A** Results from combined Nordic, United States, and Japanese patients and healthy controls. Samples from the Helsinki Biobank are included in respective groups. Dashed line denotes the statistical cutoff value determined from combined cohort data. The number of positive samples, the total number of samples as well as percentage of positive samples are given for each group. IAA immune aplastic anemia, BBANK aplastic anemia samples from biobanks, PNH paroxysmal nocturnal hemoglobinuria, MDS myelodysplastic syndrome, ICUS/CHIP idiopathic cytopenia of undetermined significance/clonal hematopoiesis of indeterminate potential, ITP idiopathic thrombocytopenia, PRCA pure red cell aplasia, LGLL large granular lymphocyte leukemia, RA rheumatoid arthritis, MS multiple sclerosis, DM1 Type 1 diabetes, Misc. AI miscellaneous autoimmune diseases, GVHD graft versus host disease, NON-AI non-autoimmune diseases. **B** Prevalence of HLA-DRB1*15:01 in aCOX-2 Ab positive (*n* = 110) and negative (*n* = 173) adult IAA patients. The *p* value for Fisher’s exact test (0.0279) is shown. **C** Platelet counts in aCOX-2 Ab positive (*n* = 115) and negative (*n* = 186) patients. **D** Treatment responses to immunosuppressive therapy (IST) and **E** eltrombopag combined with immunosuppressive therapy in aCOX-2 Ab negative and positive IAA patients. CR complete response, PR partial response, NR no response. **F** Percentages of aCOX-2 Ab positive IAA patients among HLA-DRB1*15:01 positive patients in age groups divided in 10-year intervals. **G** Follow-up samples from IAA patients (*n* = 21) were analyzed for aCOX-2 Ab over the period of up to 36 months. Immunoglobulin isotype levels were compared to the subclass means and are expressed as standard deviations (SD) from the mean. **H** Comparison of IgG, IgM and IgA in IAA patients (*n* = 24). All healthy controls (*n* = 17) were negative for all aCOX-2 Ab isotypes. **I** IgG isotypes 1–4 in aCOX-2 Ab positive (*n* = 38) cases and an aCOX-2 Ab negative control patient (*n* = 1). All tested healthy controls (*n* = 30) were negative for all IgG isotypes.

Control samples were obtained from collaborating clinical centers and from biobanks (detailed sample information in Supplementary Tables 1 and 2). All healthy controls (*n* = 74) and non-hematological patients without autoimmune conditions (*n* = 154) were tested negative for aCOX-2 Ab (Fig. 2A). Similarly, all tested patients with RA (*n* = 51), graft versus host disease (GVHD) (*n* = 56), and patients with other autoimmune diseases (*n* = 30) were aCOX-2 Ab negative, while sporadic aCOX-2 Ab positive cases were identified among multiple sclerosis (MS, *n* = 2/98, 2%) and type 1 diabetes (DM1, *n* = 2/44, 5%). Finally, we tested a patient cohort with inherited BMF diseases. These patients presented germline mutations in *RTEL* (*n* = 2), *TERT* (*n* = 6), *TERC* (*n* = 5), *FANCA* (*n* = 2), *MPL* (*n* = 1), *PARN* (*n* = 1), *GATA* (*n* = 1) genes and one patient was diagnosed with polygenic telomeropathy. All these patients were negative for the presence of aCOX-2 Ab.

Clinically, IAA shares characteristics with many related hematological disorders such as hypoplastic MDS, LGLL, and pure red cell aplasia (PRCA). All LGLL (*n* = 68) and PRCA (*n* = 12) patients were aCOX-2 Ab negative. Some aCOX-2 Ab positive cases were identified among patients with MDS (*n* = 2/80, 3%) and idiopathic thrombocytopenia (ITP, *n* = 4/105, 4%). Nineteen percent (*n* = 3/16) of PNH patients were tested aCOX-2 seropositive.

### Anti-COX-2 Ab is associated with older age, the HLA-DRB1*15:01 genotype, and lower platelet counts at diagnosis

For logistic regression analysis, we selected adult patients with <30% missing data values and with HLA genotype and treatment information available (*n* = 276). Missing data points were imputed, and the multivariate analysis revealed a significant association between HLA-DRB1*15:01 genotype and aCOX-2 Ab positivity with odds ratio (OR) reaching 14.96 (CI 6.40–34.98, *p* < 0.001) (Table 2 and Fig. 2B). Adult aCOX-2 Ab positive IAA patients were also confirmed to be older (OR 1.34, CI 1.16–1.55, *p* < 0.001) than the autoantibody negative IAA patients. The square of age had a statistically significant OR (*p* = 0.004) over one, which suggests that the probability of being aCOX-2 Ab positive increased non-linearly. There was also a statistically significant association between aCOX-2 Ab positivity and platelet count at diagnosis (OR 1.34 CI 1.16–1.55, *p* < 0.001), aCOX-2 Ab positive IAA patients displaying lower platelet counts than the aCOX-2 Ab negative IAA patients (Fig. 2C). No differences were detected between the aCOX-2 Ab positive and negative patients in disease severity or in treatment responses to IST (Fig. 2D) or eltrombopag (Fig. 2E).

**Table 2:** Logistic regression analysis of the clinical parameters (adult IAA patients *n* = 276).

	aCOX-2 Ab negative	aCOX-2 Ab positive	OR (univariable)	OR (multivariable)	OR (multivariable. MI)
HLA-DRB1*15:01					
Absent	115 (71.4)	18 (18.0)	–	–	–
Present	46 (28.6)	82 (82.0)	**11.39 (6.29–21.56.** ***p*** < **0.001)**	**11.34 (3.55–43.47.** ***p*** < **0.001)**	**14.96 (6.40–34.98.** ***p*** < **0.001)**
Gender					
Female	82 (48.5)	65 (60.7)	–	–	–
Male	87 (51.5)	42 (39.3)	0.61 (0.37–0.99. *p* = 0.048)	1.23 (0.33–4.80. *p* = 0.760)	0.77 (0.34–1.72. *p* = 0.516)
Age at dg (years)					
Mean (SD)	38.9 (17.6)	61.4 (13.7)	**1.08 (1.06–1.10.** ***p*** < **0.001)**	**1.52 (1.22–1.98.** ***p*** = **0.001)**	**1.34 (1.16–1.55.** ***p*** < **0.001)**
Age at dg^2^					
Mean (SD)	1821.9 (1636.7)	3956.6 (1612.1)	**1.00 (1.00–1.00.** ***p*** < **0.001)**	**1.00 (0.99–1.00.** ***p*** = **0.006)**	**1.00 (1.00–1.00.** ***p*** = **0.004)**
PNH clone					
Absent	78 (53.4)	41 (42.3)	–	–	–
Present	68 (46.6)	56 (57.7)	1.57 (0.94–2.64. *p* = 0.089)	1.02 (0.26–3.84. *p* = 0.981)	1.18 (0.51–2.73. *p* = 0.698)
Severity					
Moderate	41 (26.5)	28 (28.0)	–	–	–
Severe	101 (65.2)	61 (61.0)	0.88 (0.50–1.58. *p* = 0.676)	1.92 (0.27–15.57. *p* = 0.523)	1.17 (0.37–3.68. *p* = 0.782)
Very severe	13 (8.4)	11 (11.0)	1.24 (0.48–3.17. *p* = 0.654)	2.09 (0.11–43.76. *p* = 0.624)	1.49 (0.26–8.58. *p* = 0.657)
Hb at dg (g/dl)					
Mean (SD)	8.9 (2.1)	9.5 (10.1)	1.02 (0.98–1.09. *p* = 0.454)	1.36 (0.93–2.05. *p* = 0.122)	1.08 (0.90–1.29. *p* = 0.412)
WBC at dg (10^9^**/**l)					
Mean (SD)	2.4 (1.5)	2.5 (1.3)	1.05 (0.89–1.25. *p* = 0.536)	2.46 (0.83–7.64. *p* = 0.103)	1.18 (0.59–2.38. *p* = 0.637)
Plt at dg (10^9^/l)					
Mean (SD)	40.8 (46.4)	21.6 (16.8)	**0.97 (0.96–0.98.** ***p*** < **0.001)**	0.98 (0.94–1.01. *p* = 0.219)	**1.34 (1.16–1.55.** ***p*** < **0.001)**
ANC at dg (10^9^/l)					
Mean (SD)	0.9 (1.0)	0.9 (0.7)	0.90 (0.66–1.20. *p* = 0.499)	0.28 (0.06–1.13. *p* = 0.077)	0.64 (0.26–1.59. *p* = 0.336)
ALC at dg (10^9^/l)					
Mean (SD)	1.2 (0.8)	1.4 (0.7)	1.37 (0.94–2.00. *p* = 0.102)	1.08 (0.31–3.82. *p* = 0.901)	0.99 (0.38–2.55. *p* = 0.980)

Logistic regression analysis for adult (>18 years old) IAA patients. For categorical variables numbers of patients together with the proportion of patients from aCOX-2 Ab negative and positive cohorts are reported. For continuous variables mean values together with standard deviations (SD) are reported.

*Dg* diagnosis, *PNH* paroxysmal nocturnal hemoglobinuria, *Hb* hemoglobin, *WBC* white blood cells, *Plt* platelets, *ANC* absolute neutrophil counts, *ALC* absolute lymphocyte counts, *SD* standard deviation, *OR* odds ratio, *MI* multiple imputation.

Statistically significant *p*-values are in bold.

Univariate analysis of the whole cohort (*n* = 405) returned a modest, but statistically significant OR 2.02 (1.28–3.22, *p* = 0.003) for the presence of the PNH clone in the aCOX-2 Ab positive IAA patients (Supplementary Table 3). This difference, however, was not confirmed in the multivariate analyses, implying that the presence of the PNH clone is cofounded with some other variable e.g., the HLA-DRB1*15:01 genotype.

### aCOX-2 Ab assay has excellent specificity and sensitivity in older patients with HLA-DRB1*15:01 genotype

Next, IAA patients were split into HLA-DRB1*15:01 positive and negative groups. The age-dependent increase in aCOX-2 Ab positivity was even sharper when the analysis was restricted to the HLA-DRB1*15:01 positive IAA patients (Fig. 2F). Similarly, test performance indicators were markedly improved when the analysis was restricted to HLA-DRB1*15:01 positive, adult (>18 years or >40 years old) IAA patients. The overall test specificity was 98%, and sensitivity reached 83% in >40 years old HLA-DRB1*15:01 positive IAA patients (Table 3). There was also a clear improvement in in false negative rate (from 0.68 to 0.17), and in area under ROC curve (AUC, from 0.65 to 0.91) only >40 years old, HLA-DRB1*15:01 positive IAA patients were included in the analysis.

**Table 3:** Diagnostic test parameters.

	All IAA	>18 years old IAA	>40 years old IAA	All DRB1*15:01 pos IAA	>18 years old DRB1*15:01 pos IAA	>40 years old DRB1*15:01 pos IAA
Number of patients	405	334	208	174	157	113
Accuracy	0.74	–	–	–	–	–
Inaccuracy/Error rate	0.26	–	–	–	–	–
Sensitivity	0.32	0.37	0.55	0.56	0.63	0.83
95% CI	0.28–0.37	0.32–0.43	0.50–0.63	0.49–0.63	0.55–0.70	0.76–0.90
Specificity	0.98	–	–	–	–	–
95% CI	0.97–0.99	–	–	–	–	–
Youden’s index	0.30	–	–	–	–	–
False positive rate (FPR)	0.02	–	–	–	–	–
False negative rate (FNR)	0.68	0.63	0.45	0.44	0.37	0.17
Positive likelihood ratio (LR+)	16.9	–	–	–	–	–
Negative likelihood ratio (LR−)	0.69	–	–	–	–	–
Positive predictive value (PPV)	0.91	–	–	–	–	–
Negative predictive value (NPV)	0.71	–	–	–	–	–
Predictive summary index (PSI)	0.62	–	–	–	–	–
Diagnostic odds ratio (DOR)	24.4	–	–	–	–	–
Area under ROC curve (AUC)	0.65	0.68	0.77	0.77	0.81	0.91
Standard error	0.02	–	–	–	–	–
95% CI	0.61–0.69	–	–	–	–	–

Descriptive parameters to define the usefulness of aCOX-2 Ab test as a novel disease biomarker. Parameters were calculated for all IAA patients, to adult patients >18 years old and to patients >40 years of age. All age groups were analyzed both with and without restriction to the HLA-DRB1*15:01 genotype. The age and genotype information were not available for all controls, thus, many of the values are only presented for the whole cohort. Mathematical equations used for the calculations are given in Supplementary Table 4.

To confirm the persistence of aCOX-1 autoantibodies in follow-up, we measured the autoantibody levels in 21 patients who received IST as their first line treatment (Fig. 2G). In most IAA patients, the autoantibody levels remained stable during a 24–36-month follow-up. In two patients, a transient drop in the aCOX-2 Ab levels was detected at 6 months together with a partial or complete treatment response to IST. The antibody levels returned above aCOX-2 Ab assay positivity threshold by 12 months. In a 3rd patient, a similar decrease was observed at 6 months and no follow-up samples were available. In a separate cohort of nine patients, from whom follow-up samples were available at random timepoints after the initial diagnosis, aCOX-2 Ab levels were stable in all patients during the follow-up (Supplementary Fig. 4).

### The aCOX-2 Ab isotype profile is dominated by IgG1 and IgG3

The aCOX-Ab detection DELFIA assay was restricted to IgG class antibodies. To discover whether other Ig class antibodies were present, we measured aCOX-2 IgA and IgM antibody levels from 26 aCOX-2 Ab positive IAA patients and 17 healthy controls. The aCOX-2 Ab response was clearly dominated by IgG, as only three patients had both IgA and IgG antibodies and only one patient had all three isotypes (IgG, IgM, and IgA) (Fig. 2H). The triple-positive patient had an active disease during the follow-up with stably high aCOX-2 IgG levels (Supplementary Fig. 4). IgA and IgM levels were measured at the last follow-up time point (69 months from diagnosis).

Next, we studied the IgG subclass (IgG_1_–IgG_4_) distribution in aCOX-2 Ab positive IAA patients (*n* = 38), healthy controls (*n* = 30) and in one (*n* = 1) aCOX-2 Ab negative IAA patient. The negative control patient and all the healthy controls remained negative for all the tested subclasses, while all anti-COX-2 Ab positive patients presented IgG_1,_ and 55% of patients also presented IgG_3_. Only sporadic aCOX-2 IgG_2_ or IgG_4_ positive cases were detected (Fig. 2I).

### aCOX-2 autoantibodies bind the C-terminal part of COX-2

**Fig. 3:**
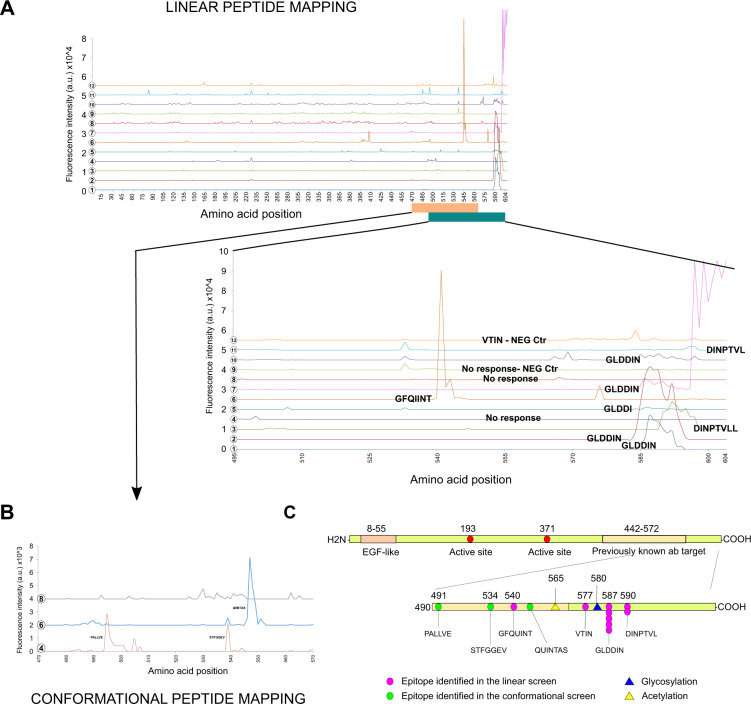
Epitope mapping. High resolution epitope mapping was performed to identify antigen epitopes from ten (*n* = 10) aCOX-2 Ab positive and from two (*n* = 2) autoantibody negative AA plasma samples. **A** Linear 15 amino acid peptides with 14 amino acid overlap covering the whole COX-2 protein were spotted on a microchip. Antibody binding to the linear peptide was detected with goat anti-human IgG (Fc) DyLight6 and LI-COR Odyssey Imaging System. Scanning values are reported as fluorescence intensities (a.u.). **B** Autoantibody samples from which no clear epitope could be identified using linear peptides, were subjected for conformational peptide mapping. Here all peptides were cyclized using a thioether linkage linking the amino and carboxy terminals. The peptides were spotted on a microarray as 10-mers (7 and 13-mers presented in Supplementary Fig. 4) with a peptide-peptide overlap of n-1 amino acids. Signal intensities are reported as fluorescence intensities (a.u.). **C** Visual summary of all epitope findings reveals a shared antigenic protein sequence that is localized between amino acids 490 and 590 in the C-terminal part of COX-2.

Denaturing SDS-PAGE electrophoresis followed by western blotting was performed with patients’ plasma samples, and it confirmed that in nine out of ten cases, aCOX-2 antibodies were able to bind denatured full-length recombinant COX-2 (Supplementary Fig. 5). To reveal the antigenic epitope in COX-2, a microarray-based linear peptide mapping platform was utilized. We identified an almost identical, DIN amino acid (D590-N592) signature containing C-terminal epitope in five of the ten tested aCOX-2 Ab positive patient plasma samples and additionally, other closely mapping C-terminal epitopes in two patients (Fig. 3A). The linear mapping method could not identify an antigenic epitope for three of the aCOX-2 Ab positive patients, and thus, they were subjected for conformational peptide screen (Fig. 3B and Supplementary Fig. 6). Conformational peptide mapping with cyclic peptides revealed two additional C-terminal COX-2 epitopes. All the identified epitopes are summarized in Fig. 3C.

### The expression of *PTGS2* mRNA

**Fig. 4:**
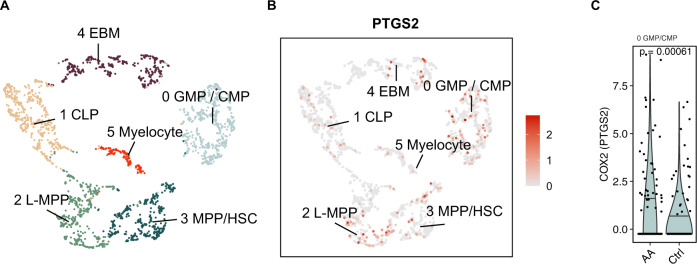
*COX-2* expression in healthy controls’ and in AA patients’ bone marrow samples. **A** UMAP representation of single-cell RNA-sequencing profiles from 15 untreated AA patients and 2 healthy bone marrow samples and (data reanalyzed from Zhu et al. [33]), colored by cell cluster. **B** *COX-2* (*PTGS2*) expression over the cell phenotypes. **C** Differential expression of *COX-2* (*PTGS2*) in GMP/CMP cells from untreated AA patients and healthy donors. *p* value was calculated with a two-sided Mann–Whitney test. EBM eosinophil, basophil, and mastocyte progenitor, GMP/CMP granulocyte-monocyte precursor/common myeloid precursor, CLP common lymphoid precursor, L-MPP lymphoid-primed multipotent progenitor, MPP/HSC multipotent progenitor/hematopoietic stem cell.

To understand in which cells *PTGS2* (gene coding for COX-2) is expressed in human bone marrow, we reanalyzed a recently published single-cell RNA-sequencing (scRNAseq) data set [33] including CD34+ cells from bone marrow samples from untreated AA patients (*n* = 15) and healthy controls (*n* = 2). Of the identified cell phenotypes (Fig. 4A), The highest *PTGS2* expression levels were found in the granulocyte-monocyte/common monocyte precursor (GMP/CMP) and multipotent-progenitor /hematopoietic stem cell (MPP/HSC) clusters (Fig. 4B). Interestingly, the *PTGS2* expression levels were significantly elevated in AA patients’ GMP/CMP cluster (Fig. 4C).

## Discussion

Autoantibody measurements are commonly used diagnostic tests in many autoimmune diseases such as RA [38], celiac disease [39], and autoimmune hemolytic anemia [40]. In IAA, some previous autoantibody candidates have been identified, but their clinical use has been hampered with relatively poor specificity and sensitivity. We screened new autoantibody candidates for IAA using microarray technology covering >9000 full-length proteins and identified a novel autoantibody against COX-2, which was present in 37% of adult patients with IAA (project results summarized in Supplementary Fig. 7). The overall specificity of aCOX-2 ab for IAA was 98%, and in older patients (>40 years of age) with the HLA-DRB1*15:01 genotype, 83% sensitivity was achieved.

The high specificity of aCOX-2 Ab is further underscored by the fact that no aCOX-2 Ab seropositive individuals were identified among the healthy controls or patients with constitutive BMF diseases. Also, all patients with rheumatic diseases, GVHD, LGLL and PRCA were aCOX-2 Ab seronegative. Two aCOX-2 Ab positive patients were identified among the MS and T1D cohorts. As the clinical presentation of IAA is completely different from these diseases, the finding does not directly impact the usefulness of the autoantibody as a clinically relevant disease biomarker. Interestingly, MS is also associated with the DRB1*15:01 [41] genotype, which may, at least in part, explain the aCOX-2 Ab positive cases in the MS cohort.

Unlike the previously reported IAA-associated autoantibodies against DRS-1 [23], CLIC1, HSP11 and RSP27 [27], aCOX-2 Ab was not commonly detected in MDS. Two MDS patients were tested aCOX-2 Ab positive, and they both presented with hypoplastic MDS, which implies that there may be common disease mechanisms operating in hypoplastic MDS and IAA, or these two cases may have been misdiagnosed. Moesin [28], CA-1 [24, 25], hnRNP K [28] and DRS-1 [23] are previously reported autoantibody targets in IAA which were also included in our microarray screening platform. None of our discovery cohort patients showed detectable reactivity against these previously reported self-antigens.

aCOX-2 autoantibody prevalence varied between the three examined patient cohorts. The highest degree of seropositivity was discovered in the Nordic IAA cohort (61 %), while the prevalence was markedly lower in the US (26 %) and Japanese (37 %) IAA cohorts. The positivity rates were directly comparable with the age distributions in the different cohorts—the US cohort being youngest and the Nordic cohort being the oldest. We also examined the possible connection between the cohorts’ seropositivity rates and population frequencies of the HLA-DRB1*15:01 genotype. The lower population frequency of HLA-DRB1*15:01 in the Japanese (5–9%) compared to the Caucasian (15%) population (allelefrequencies.net, retrieved 17th Jan 2020) fit well with the difference in the seropositivity rate between the Nordic and Japanese IAA patients, but does not explain the difference between the US and Nordic cohorts as they both primarily consisted of Caucasian patients.

The immune-mediated destruction of HSCs in AA is commonly related to cytotoxic T cells [1]. The role of other immune cell subtypes, more specifically B cells, has not been well characterized. Previous research has shown that the number of circulating B cells in IAA patients is similar to what is seen in healthy individuals [42]. Immunosuppressive drugs, such as cyclosporin, are known to inhibit B-cell proliferation [43]. However, autoantibodies are produced by long lived plasma cells which function is less likely to be affected by non-B-cell depleting [44]. In concord, aCOX-2 Ab levels were stable during follow-up, and only in incidental cases the levels dropped slightly during the first 6 months of successful therapy but returned high as the disease relapsed. The biochemical properties of antibodies are known to regulate different immunological effector mechanisms, and IgG_1_ and IgG_3_ isotypes, which were also dominant forms of aCOX-2 autoantibodies, can activate the complement system [45]. Similarly as in our study, autoantibodies in SLE follow the same isotype distribution pattern with IgG_1_ and IgG_3_ as the predominant isotypes [46]. The antibody production is not only dependent on B cells, but initially requires MHC II driven antigen presentation to T helper cells. The HLA-DRB1*15:01 association of aCOX-2 Ab connects the autoantibody findings also to T cell responses. HLA-DRB1*15:01 has previously been shown to be overrepresented in AA patients and associates with favorable treatment responses to immunosuppression [47], and with the presence of the PNH clone [48]. Both the HLA-association, autoantibody isotype distribution, and strong age dependency encourage to address the biological significance of the aCOX-2 autoantibodies in the IAA pathogenesis in the future.

aCOX-2 Ab positive IAA patients had significantly lower platelet counts at diagnosis when compared to autoantibody negative patients. Interestingly, sporadic aCOX-2 Ab positive cases were also noted among patients with chronic ITP. Previously, *PTGS-2* expression has been shown to be induced during human megakaryopoiesis [49]. The importance of COX-2 for platelets is further supported by defective megakaryopoiesis and platelet function in COX-2 deficient mice [50]. Eltrombopag, a small molecule agonist of the trombopoietin receptor, has shown good clinical efficacy in IAA [51–53]. In our study, we had a small cohort of patients (*n* = 21) treated with eltrombopag and IST combination regimen. No clear differences in the treatment responses between autoantibody positive and negative patients were observed. As the median age in this patient cohort was low (41 years), and only 13 aCOX-2 Ab positive (diagnostic, non-treated) patients were selected for the analysis, the results should be evaluated with caution. Follow-up studies evaluating the impact of autoantibody on the treatment responses in elderly patients receiving eltrombopag as a monotherapy are warranted.

Taken together, the aCOX-2 Ab defines a distinct group of IAA and has the potential for a clinical disease biomarker, as it has good sensitivity and specificity, and it is cheap and easy to perform using pre-existing technical and logistic platforms in clinical laboratories. The strong age and HLAII association warrant further studies to clarify whether aCOX-2 Abs contribute to IAA pathogenesis or whether they develop as an epiphenomenon after the tolerance against the blood forming tissue is broken.

## Supplementary information

Supplemental material
