# Supplementary material for: Anti-COX-2 autoantibody is a novel biomarker of immune aplastic anemia
Source: Leukemia. 2022 Aug 5;36(9):2317–27. doi: 10.1038/s41375-022-01654-6 (PMC9417997; doi:10.1038/s41375-022-01654-6)
Supplement: Supplementary file 1 — Supplemental material [file 41375_2022_1654_MOESM1_ESM.pdf]

Supplementary Materials

Table of contents 1

Supplementary Figures

Supplementary Figure 1. Determining cutoff for positivity 2
Supplementary Figure 2 Missing clinical data values 3
Supplementary Figure 3. Replication of microarray data with DELFIA 4
Supplementary Figure 4. Follow-up samples from the Finnish cohort 5
Supplementary Figure 5. Epitope summary and SDS-PAGE Electrophoresis 6
Supplementary Figure 6. Conformational peptide screen 7
Supplementary Figure 7. Summary of project workflow and results 8

Supplementary Tables

Supplementary Table 1. Control cohorts from clinical centers 9
Supplementary Table 2. Helsinki Biobank patient cohort 10
Supplementary Table 3. Logistic regression – all IAA patients 11
Supplementary Table 4. Formula for calculation of different test characteristics 12

Supplementary Methods 13

Supplementary Figure 1

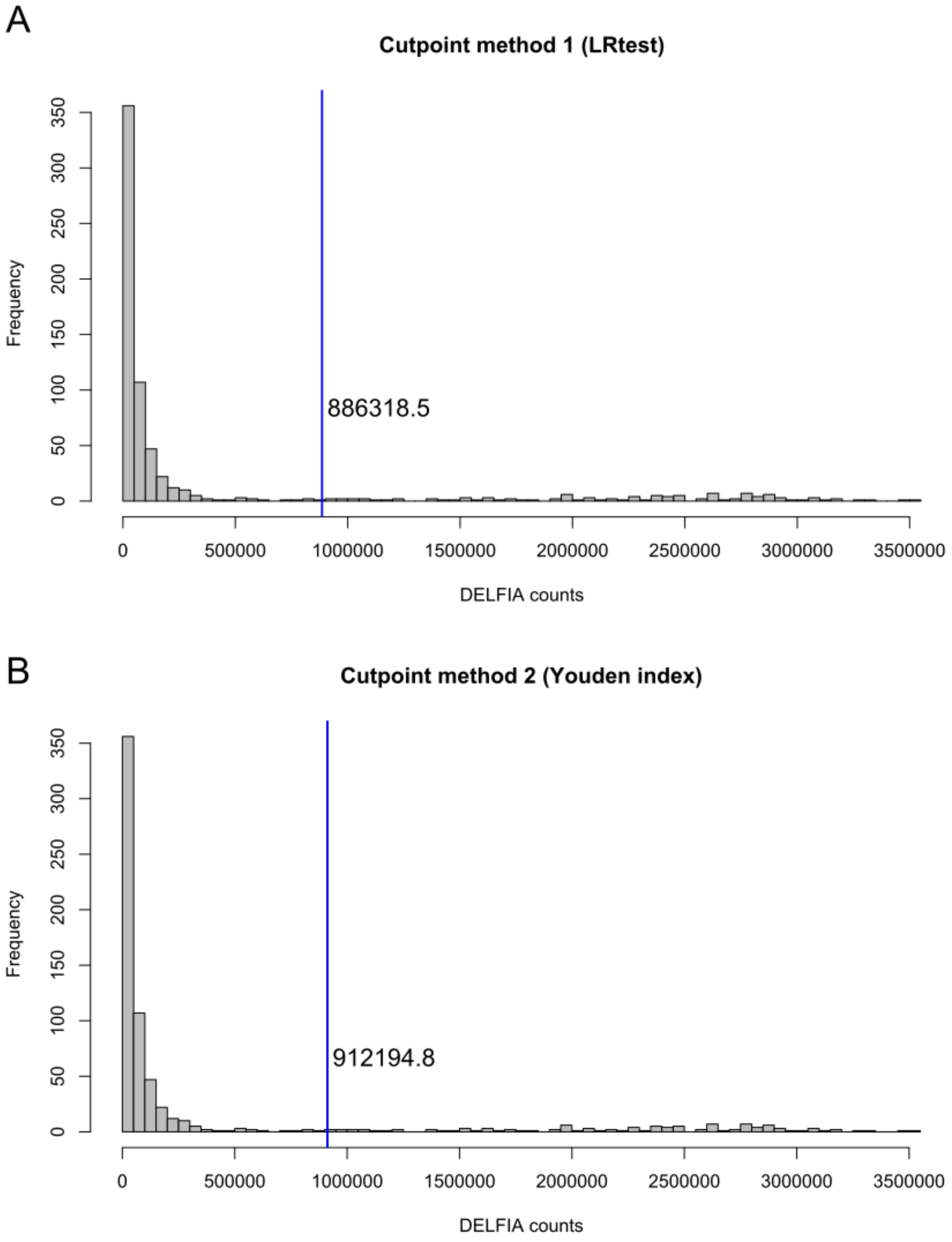

Supplementary Figure 1. Determining cutoff for positivity. The training data presented in histogram was combined cohorts of n = 681 patients, blue line with value denotes the optimal cutoff with DELFIA counts predicting the presence of AA. (A) Using method Findcutoffs, minimizing likelihood ratio test p-value (p = 7.43e-32) and maximizing AUC in ROC curve (0.659). (B) Using method OptimalCutPoint, maximizing Youden's *J* statistic (3.19e-01).

Supplementary Figure 2

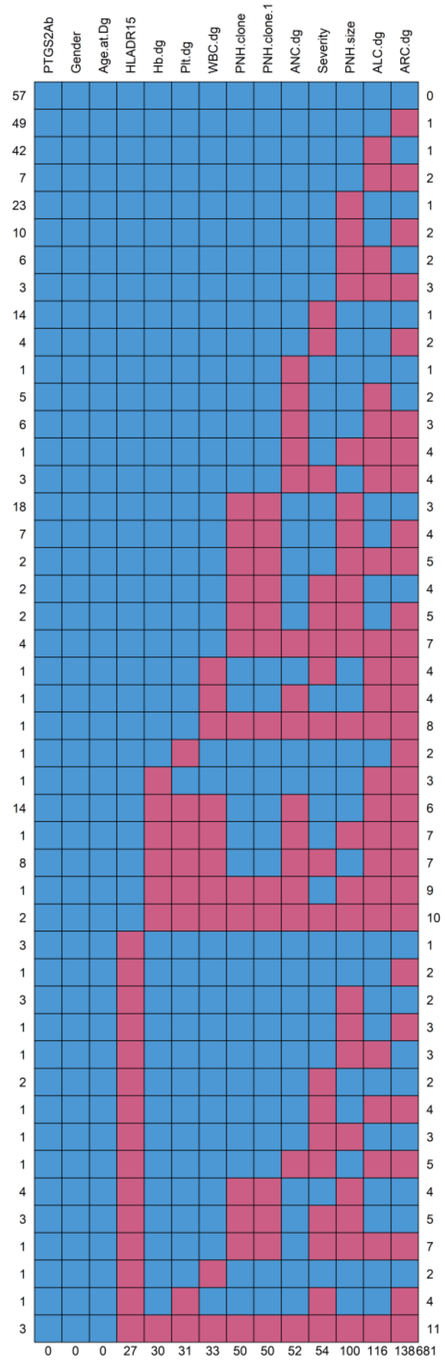

Supplementary Figure 2. Missingness patterns in the clinical data from adult (>18 years old) IAA patients (n=334). Blue color indicates reported variables, red color indicates missing variables. Numbers in the left indicate the number of patients who have data for all blue variables. Numbers on the right side indicate the number of patients with data missingness pattern corresponding the red squares.

Abbreviations: PTGSAb = aCOX-2 Ab, HLADR15 = Presence of HLA-DRB15*15:01, PNH clone = Presence of the PNH clone – divided into clinical and sub-clinical, PNH clone.1 = Presence of the PNH clone, ANC = Absolute neutrophil count, ALC = Absolute lymphocyte count, ARC = Absolute reticulocyte count.

Supplementary Figure 3

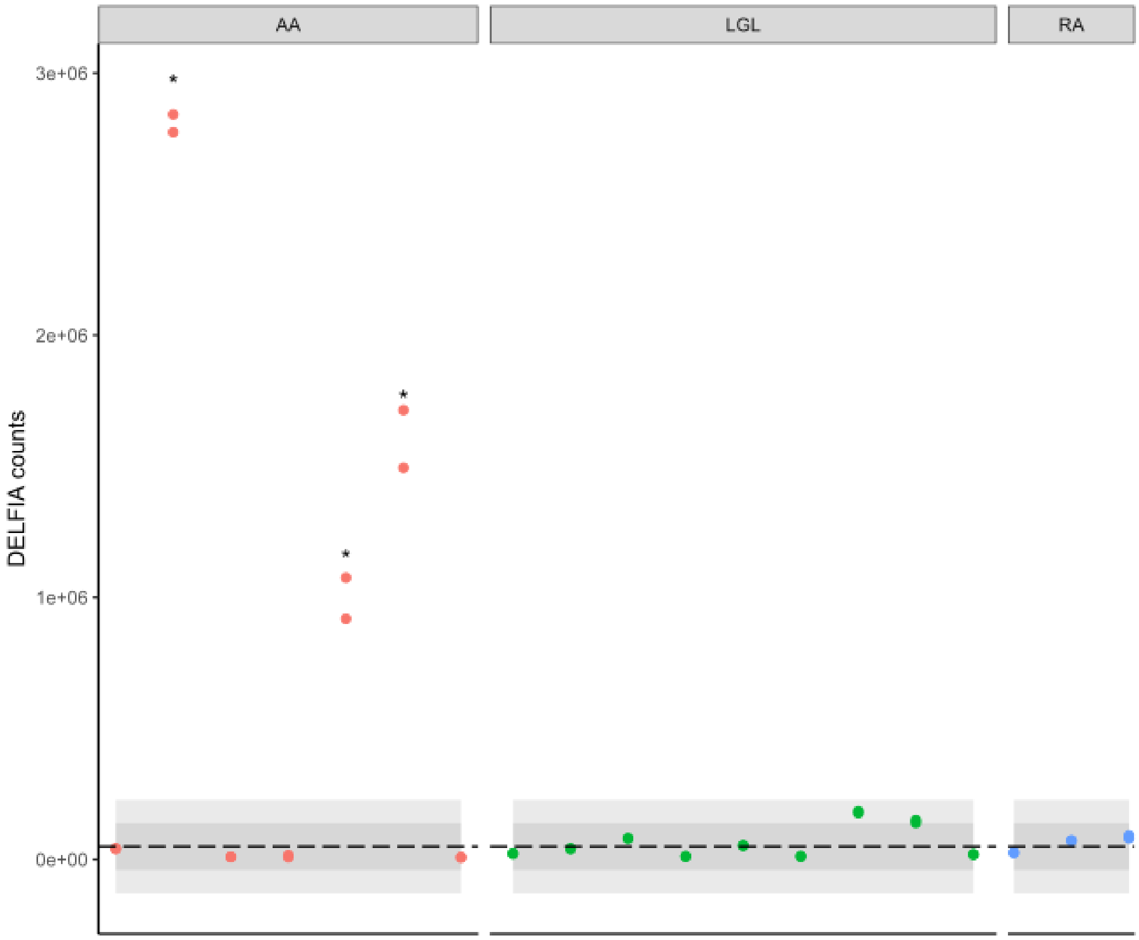

Supplementary Figure 3. Replication of microarray data with DELFIA. Points are duplicate measurements in DELFIA for n = 7 aplastic anemia patients (AA), n = 9 LGL leukemia patients (LGL) and n = 3 rheumatoid arthritis patients (RA). For negative patients the duplicate points partially overlap. All cases here were also included in the microarray. Dashed line = healthy controls (HC) mean in DELFIA (n = 30), dark grey area = ±1 SD’s of mean HC, light grey area = ±2 SD’s of mean HC, * = denotes positive cases found positive on microarray assay.

Supplementary Figure 4

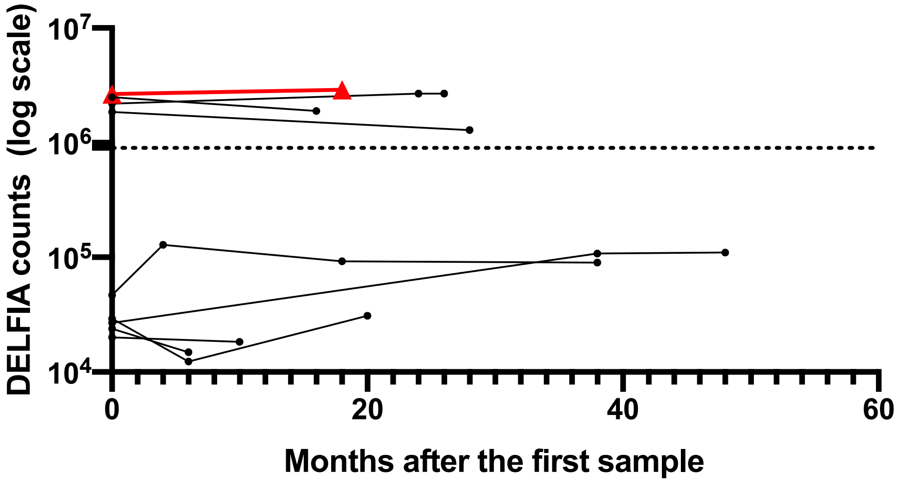

Supplementary Figure 4. Follow-samples from the Finnish cohort. Patient samples were collected at different time points after the initial diagnosis was set. All patients from which we obtained serial samples retained their aCOX-2 antibody status in follow-up. Timeline from the first obtained sample is presented on the x-axis. The dashed vertical line represents the cut-off for aCOX-2 Ab positivity. Red symbols indicate the patient, who presented with an aberrant immunoglobulin subclass distribution with IgG, IgM and IgA present.

Supplementary Figure 5

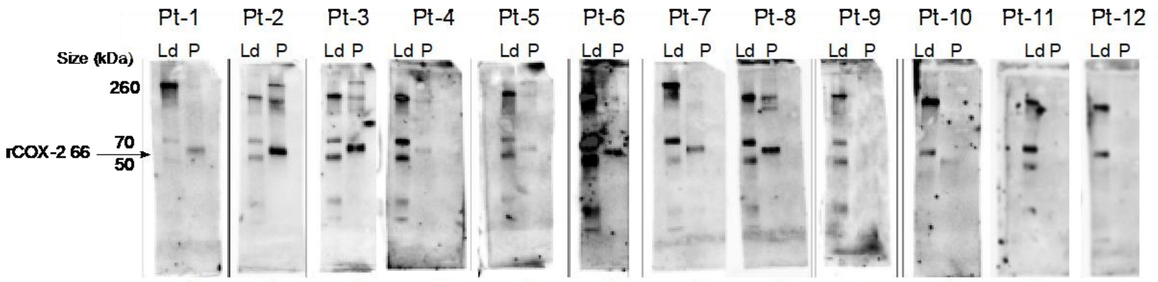

	Pt-1	Pt-2	Pt-3	Pt-4	Pt-5	Pt-6	Pt-7	Pt-8	Pt-9	Pt-10	Pt-11	Pt-12
DELFIA	+	+	+	+	+	+	+	+	-	+	+	-
WB	+	++	++	+	+	++	++	++	-	+	-	-
C-TERMINAL EPITOPE	++	++	++	-	++	-	+	-	-	++	+	-
CONFORM. EPITOPE	ND	ND	ND	+	ND	+	ND	-	ND	ND	ND	ND
HLA-DRB1*15:01	+	+	+	+	ND	+	+	+	+	+	-	-

Supplementary Figure 5. Epitope summary and SDS-PAGE Electrophoresis. Membrane-bound recombinant COX-2 was probed with IAA patient plasma samples (n=12) with known aCOX-2 Ab status determined with DELFIA sandwich assay. Molecular weight marker is shown for each patient sample. All autoantibody-positive plasma samples from IAA patients with known HLA-DRB1*15:01 positive genotype bound recombinant COX-2 also in its linearized form. Plasma sample from an autoantibody positive, but HLA-DRB1*15:01 negative did not give positive signal in WB against rCOX-2.

Abbreviations: Pt, patient; Ld, ladder molecular weight marker; P, plasma sample; WB, western blot, ND, not done.

In the Delfia analysis autoantibody positive patients are marked with +.
In the WB analysis ++ denotes strong positive signal and + weaker positive signal.
In the linear epitope analysis ++ denotes strong signal from the consensus sequence in the C-terminal part of rCOX2 and + weaker signal of target outside from the consensus region.
In the conformational epitope analysis + denotes conformational epitope identified close to the C-terminal.

The presence of the HLA-DRB1*15:01 genotype is marked with +.

Supplementary Figure 6

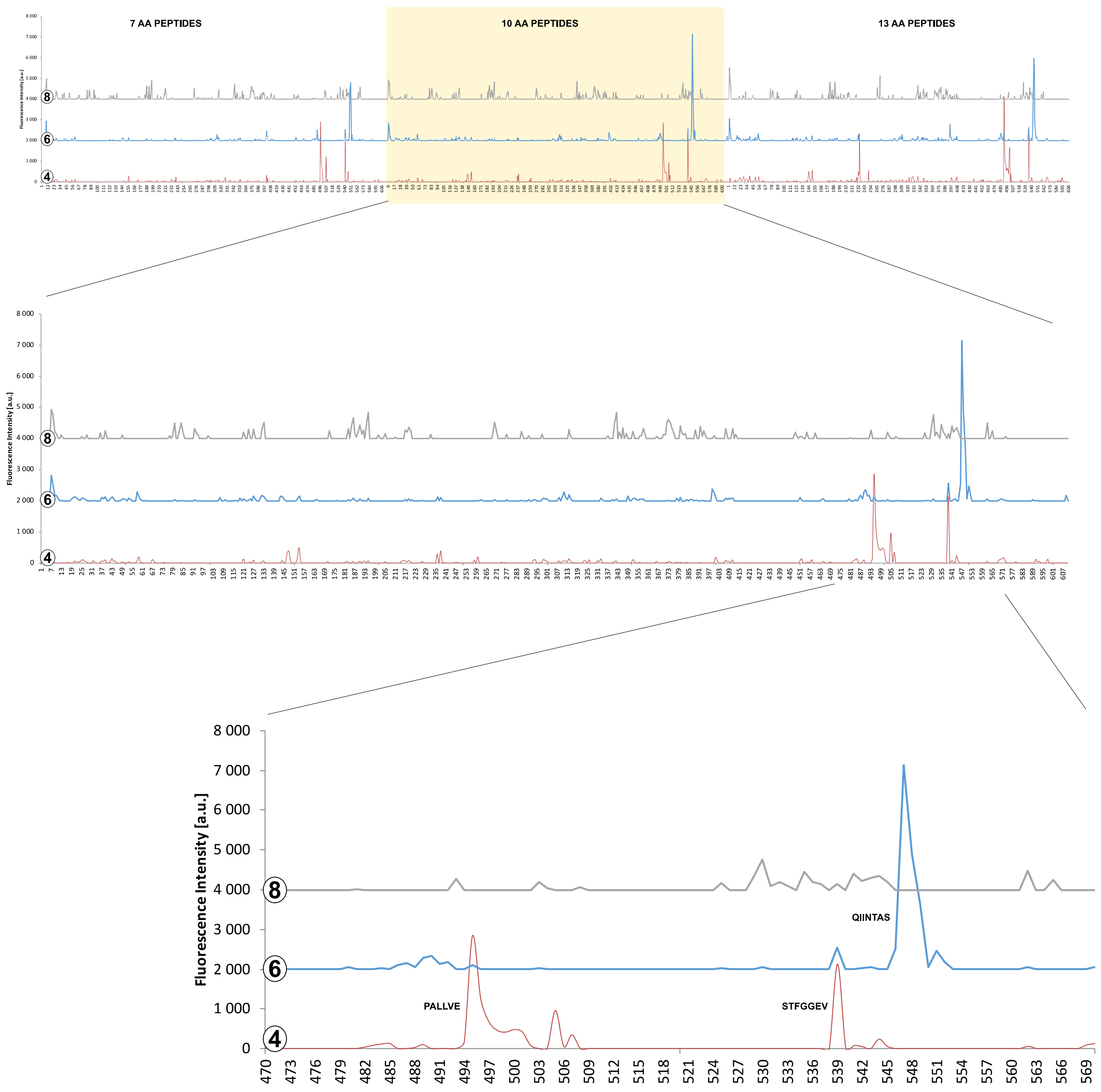

Supplementary Figure 6. Conformational peptide screen. The analysis was performed using cyclic, 7-, 10- and 13-mer peptides. Co parable epitopes were identified using all peptide lengths. Data obtained using the 10-mers is zoomed in to the COX-2 protein antigenic epitopes residing between amino acids 490 and 555.

Supplementary Figure 7

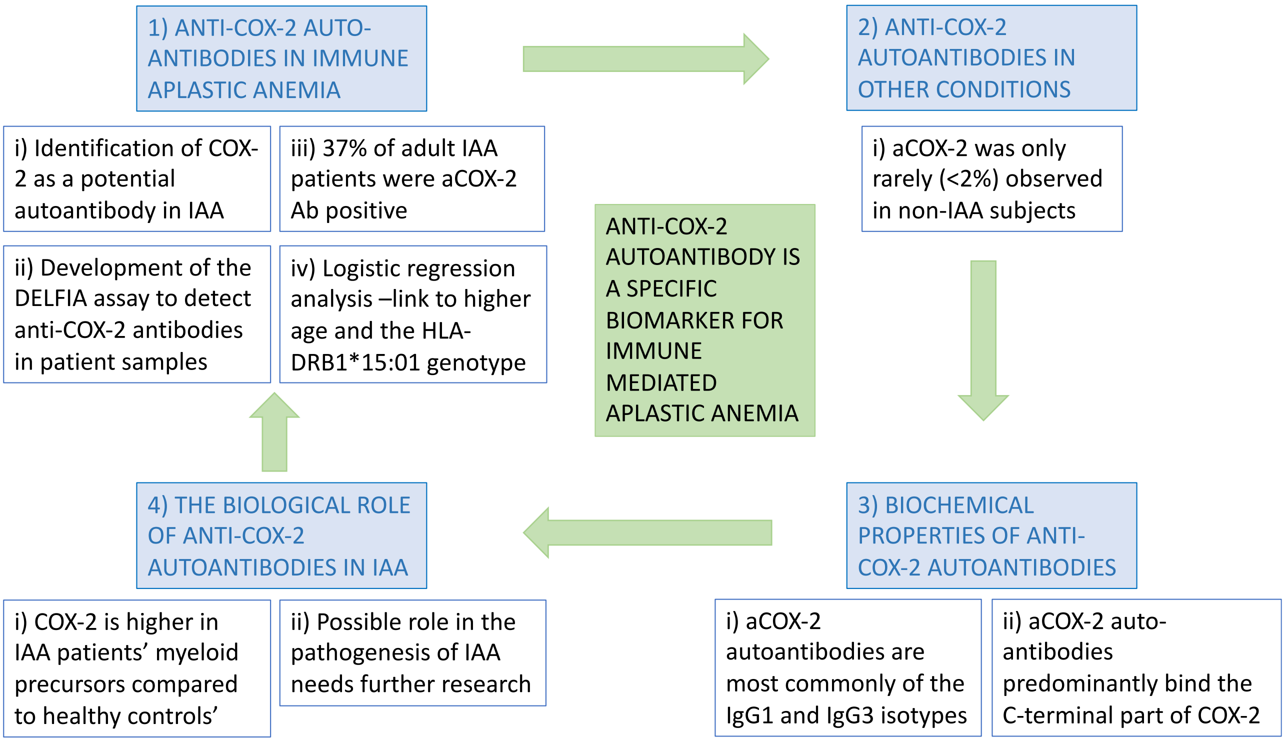

Supplementary figure 7. Summary of the project workflow and results

Supplementary Tables

Supplementary Table 1. Control cohorts

Diagnosis	Number of Patients	NORD	US	JPN	HBB	FHRB
IAA (>18 years)	334	37	209	88	-	-
IAA (<18 years)	52	1	46	5	-	-
IAA (age not known)	19	-	4	15	-	-
HBB AA	12	-	-	-	12	-
FHRB AA	9	-	-	-	-	9
PNH	16	1	13	2	-	-
MDS	80	16	15	34	17	-
ICUS/CHIP	8	8	-	-	-	-
C-BMF	19	0	19	0	0	-
ITP	105	24	10	16	55	-
PRCA	12	-	-	12	-	-
LGLL	68	49	19	-	-	-
RA	51	50	-	-	1	-
MS	98	-	-	-	98	-
DM1	44	-	-	-	44	-
Misc.AI	30	-	-	-	30	-
GVHD	56	56	-	-	-	-
NON-AI	154	-	2	-	152	-
Healthy	74	30	24	20	-	-
**Total**	**1241**	**272**	**361**	**192**	**409**	**9**

Supplementary Table 1. Control cohorts

Abbreviations: NORD, Samples from the Nordic countries (mostly Finland), BBANK AA, Aplastic anemia samples from Helsinki Biobank (HBB) and from The Finnish Hematology Registry and Clinical Biobank (FHRB Biobank); IAA, immune aplastic anemia; PNH, paroxysmal nocturnal hemoglobinuria; MDS, myelodysplastic syndrome; ICUS/CHIP, idiopathic cytopenia of undetermined significance/ clonal hematopoiesis of indeterminate potential; ITP, idiopathic thrombocytopenia; PRCA, pure red cell aplasia; LGLL, large granular lymphocyte leukemia; RA, rheumatoid arthritis; MS, multiple sclerosis; DM1, Type 1 diabetes; Misc. AI, miscellaneous autoimmune diseases; GVHD, graft versus host disease; NON-AI, non-autoimmune diseases

Supplementary Table 2. Helsinki Biobank patient cohort

Explanation	Number of Patients	Group
Myelodysplastic syndromes	17	MDS
Autoimmune or unspecified hemolysis	2	Misc. AI
Hemolytic uremic syndrome	1	NON-AI
Aplastic anemia	12	HBP IAA
Neutropenia (unspecified)	20	NON-AI
Sarcoidosis	2	NON-AI
Immunoglobulinopathy	4	NON-AI
Thyroid diseases	2	NON-AI
Diabetes mellitus type 1	44	DM1
Electrolyte problem	2	NON-AI
Multiple sclerosis	98	MS
Vasculitis (all types)	15	NON-AI
Inflammatory bowel disease	2	Misc.AI
Unspecified hepatic disease	1	NON-AI
Autoimmune hepatitis	1	Misc.AI
Malabsorption (unspecified/intolerance)	1	NON-AI
Rheumatoid arthritis	1	RA
Idiopathic thrombocytopenia	55	ITP
Spondyloarthropathies	1	NON-AI
Arthrosis, any non-immunologic	5	NON-AI
Systemic lupus erythematosus	18	Misc.AI
Unspecified rheumatic diseases	9	Misc.AI
Systemic connective tissue diseases together	19	NON-AI
Vertebral/medullar/radicular problems	3	NON-AI
Infection	25	NON-AI
Unspecified tumor, malign and benign	31	NON-AI
Psychiatric diseases	4	NON-AI
Pneumothorax	1	NON-AI
Neurologic and intracranial diseases	6	NON-AI
Rhinitis	1	NON-AI
Diabetes mellitus type 2	1	NON-AI
Metabolic disorders	2	NON-AI
Unspecified gland diseases	1	NON-AI
Eye or auxilliary	1	NON-AI
Irritable bowel syndrome	1	NON-AI
**Total**	**409**	

Supplementary Table 3.

		aCOX-2 Ab negative	aCOX-2 Ab positive	OR (univariable)	OR (multivariable)
HLA-DRB*15:01	Absent	167 (89.3)	20 (10.7)	-	-
	Present	79 (43.9)	101 (56.1)	**10.68 (6.28-18.92, p<0.001)**	**6.38 (2.51-17.59, p<0.001)**
Gender	Female	144 (66.1)	74 (33.9)	-	-
	Male	132 (70.6)	55 (29.4)	0.81 (0.53-1.23, p=0.329)	1.44 (0.54-3.99, p=0.465)
Age at dg	Mean (SD)	34.3 (19.5)	60.7 (14.9)	**1.08 (1.06-1.09, p<0.001)**	**1.09 (1.06-1.12, p<0.001)**
PNH clone	Absent	125 (74.4)	43 (25.6)	-	-
	Present	102 (59.0)	71 (41.0)	**2.02 (1.28-3.22, p=0.003**)	1.12 (0.42-3.02, p=0.824)
Hb at dg (g/dl)	Mean (SD)	8.9 (2.1)	9.5 (9.6)	1.01 (0.97-1.07, p=0.468)	1.10 (0.81-1.51, p=0.532)
WBC at dg (10^9/l)	Mean (SD)	2.5 (1.6)	2.5 (1.2)	1.01 (0.86-1.17, p=0.929)	1.61 (0.68-3.69, p=0.267)
Plt at dg (10^9/l)	Mean (SD)	41.2 (44.7)	20.8 (16.4)	**0.97 (0.96-0.98, p<0.001)**	**0.97 (0.94-0.99, p=0.024)**
ANC at dg (10^9/l)	Mean (SD)	0.9 (1.0)	0.8 (0.7)	0.88 (0.66-1.14, p=0.337)	0.36 (0.11-1.07, p=0.070)
ALC at dg (10^9/l)	Mean (SD)	1.3 (0.8)	1.4 (0.7)	1.17 (0.84-1.63, p=0.332)	1.14 (0.44-2.95, p=0.786)

Supplementary Table 3. Logistic regression – all IAA patients (n=405). For non-parametric variables numbers of patients together with (percentages of row totals) are reported. For parametric tests mean values together with (standard deviations, SD) are reported. PNH = paroxysmal nocturnal hemolysis. Hb = hemoglobin. WBC = white blood cells. Plt = platelets. ANC = absolute neutrophil counts. ALC = absolute lymphocyte counts. SD = standard deviation. OR = odds ratio. MI = multiple imputation.

Supplementary Table 4.

	Formula
**Accuracy**	(a+d)/(a+b+c+d)
**Inaccuracy / Error rate**	1-accuracy
**Sensitivity (sens)**	a/(a+c)
**95 % confidence interval**	
**Specificity (spec)**	d/(b+d)
**95 % confidence interval**	
**Youden's Index**	(sens+spec)-1
**False positive rate (FPR)**	b/(b+d)=1-spec
**False negative rate (FNR)**	c/(a+c)=1-sens
**Positive likelihood ratio (LR+)**	sens/(1-spec)
**Negative likelihood ratio (LR)**	(1-sens)/spec
**Positive predictive value (PPV)**	a/(a+b)
**Negative predictive value (NPV)**	d/(c+d)
**Predictive summary index (PSI)**	PPV+NPV-1
**Diagnostic odds ratio (DOR)**	(a/c)/(b/d)

****

		True status	
		**Condition present**	**Condition absent**
**Test outcome**	**Positive**	(a) True positive	(b) False positive
	**Negative**	(c) False negative	(d) True negative

Supplementary Table 4. Formula for calculation of different diagnostic test characteristics.

Supplementary Methods

Linear epitope mapping

Both linear and conformational epitopes were mapped using the PEPperPRINT® technology. The sequence of prostaglandin G/H synthase 2 (UniProt ID P35354) was elongated with neutral GSGSGSG linkers at the C- and N-terminus to avoid truncated peptides. The elongated antigen sequence was translated into linear 15 amino acid peptides with a peptide-peptide overlap of 14 amino acids for high-resolution epitope mapping. The prostaglandin G/H synthase 2 peptide microarrays contained 604 different peptides printed in duplicate (1,208 peptide spots) and were framed by additional HA (YPYDVPDYAG) and polio (KEVPALTAVETGAT) control peptides (44 peptide spots each control).

Plasma dilutions of 1:500 and 1:100 were incubated and the signal was detected with goat anti-human IgG (Fc) DyLight680 (0.1 µg/ml) secondary antibody. Mouse monoclonal anti-HA (12CA5) DyLight800 (0.5 µg/ml) was used as control antibody. Measurements were made with LI-COR Odyssey Imaging System; scanning offset 0.65 mm, resolution 21 µm, scanning intensities of 7/7 (red = 700 nm/green = 800 nm).

Conformational epitope mapping

The elongated antigen sequence was translated into 7, 10 and 13 amino acid peptides with a peptide-peptide overlap of 6, 9 and 12 amino acids. After peptide synthesis, all peptides were cyclized via a thioether linkage between a C-terminal cysteine and an appropriately modified N-terminus. The conformational prostaglandin G/H synthase 2 peptide microarrays contained 1,827 different peptides printed in duplicate (3,654 peptide spots) and were framed by additional HA (YPYDVPDYAG, 64 spots) and polio (KEVPALTAVETGAT, 62 spots) control peptides. Detection as in linear epitope mapping.
